# Very-high-risk (VHR) localized prostate cancer: an indication for multimodal therapy

**DOI:** 10.18632/oncotarget.26757

**Published:** 2019-03-08

**Authors:** Debasish Sundi, Brian F. Chapin

**Affiliations:** Department of Urology, The Ohio State University Comprehensive Cancer Center, Columbus, Ohio, USA; Department of Urology, The University of Texas M.D. Anderson Cancer Center, Houston, Texas, USA

**Keywords:** prostate cancer, risk stratification, metastasis, survival

Risk stratification in localized prostate cancer (PCa) has come a long way since the seminal 1998 publication by D'Amico *et al*. that described how biochemical recurrence rates after treatment vary according to clinical risk group [1]. Notable examples of these risk stratification tools include the Partin tables (for pathologic stage) [2], the pre-operative Kattan nomogram (initially developed for biochemical recurrence) [3], the Briganti nomogram (for pathologic nodal metastases) [4], the CAPRA score (for biochemical recurrence, metastasis, and cancer-specific survival) [5], magnetic resonance imaging (for pathologic stage) [6], and genomic assays (such as Decipher, for metastasis) [7]. These prognostic tools are useful for individual patient counseling, but enrollment into clinical registries or trials is still based largely on the basic D'Amico risk structure: low, intermediate, and high. Within each of the three major risk groups, there is considerable variability in cancer outcomes.

Specifically, some high-risk prostate cancers are curable with a single treatment modality, while others will rapidly recur, metastasize, and potentially lead to cancer-specific mortality despite initial treatment with curative intent. We began a series of studies on very-high-risk (VHR) prostate cancer with the idea that the high-risk group might be able to be dichotomized based on inflection points in basic clinical features (disease stage, PSA, and biopsy results) that are associated with metastasis and cancer-related deaths. Our vision is that the resulting VHR criteria, which identify men with the most-aggressive prostate cancers, might serve as optimal enrollment criteria for clinical trials that evaluate combination therapies; since patients with VHR prostate cancer would have the most potential clinical benefit.

In a discovery cohort of 753 men with localized high-risk (clinical stage T3-4 or PSA >20 ng/ml or Gleason sum 8-10) PCa undergoing radical prostatectomy, we noted that three specific factors identified men who bore the highest burden of metastasis and cancer-specific mortality: primary Gleason pattern 5 present on biopsy, or five or more biopsy cores containing Gleason sum 8-10, or presence of multiple high-risk features [8]. These same factors, the VHR criteria, was found to be similarly prognostic in a validation study of 1981 men who underwent radical prostatectomy for high-risk PCa [9]. Notably, despite the relative simplicity of VHR criteria, they were more closely associated with prostate-cancer related deaths in these study populations than either the Kattan or CAPRA nomograms.

Considering that the 5-year event rates for metastasis and cancer-specific mortality (19.5% and 4.5%, respectively) for VHR men were considerably higher than for men with non-VHR, high-risk disease (7.6% and 0.6%, respectively), men with VHR are likely to be ideal candidates for novel combination therapies that may increase cure rates. Moreover, it is oncologically safe to enroll men with VHR PCa into clinical trials. Reichard *et al*. analyzed if time/delay to standard treatment was associated with disease recurrence, metastasis, or survival; and found that cohorts of men with VHR prostate cancer who underwent radical prostatectomy <8 weeks, 8-12 weeks, or >12 weeks from diagnosis had similar outcomes [10]. The impetus has never been clearer: men with VHR prostate cancer suffer unusually aggressive oncologic outcomes and should be considered for multimodal treatment approaches, preferably in the context of clinical trials.
